# Mid-term outcomes of Sapien 3 versus Perimount Magna Ease for treatment of severe aortic stenosis

**DOI:** 10.1186/s13019-020-01203-1

**Published:** 2020-06-29

**Authors:** Marko P. O. Virtanen, Markku Eskola, Mikko Savontaus, Tatu Juvonen, Matti Niemelä, Teemu Laakso, Annastiina Husso, Maina P. Jalava, Tuomas Tauriainen, Tuomas Ahvenvaara, Pasi Maaranen, Eeva-Maija Kinnunen, Sebastian Dahlbacka, Mika Laine, Timo Mäkikallio, Antti Valtola, Peter Raivio, Stefano Rosato, Paola D’Errigo, Antti Vento, Juhani Airaksinen, Fausto Biancari

**Affiliations:** 1 Heart Hospital, Tampere University Hospital, Tampere, Finland; 2 Faculty of Medicine and Health Technology, University of Tampere, Tampere, Finland; 3 Heart Center, Turku University Hospital, and Department of Surgery, University of Turku, Turku, Finland; 4 Heart and Lung Center, Helsinki University Hospital, Helsinki, Finland; 5 Department of Surgery, Oulu University Hospital and Research Unit of Surgery, Anesthesia and Intensive Care, Faculty of Medicine, University of Oulu, Oulu, Finland; 6 Department of Internal Medicine, Oulu University Hospital, Oulu, Finland; 7 Heart Center, Kuopio University Hospital, Kuopio, Finland; 8 National Centre of Global Health, Istituto Superiore di Sanità, Rome, Italy

**Keywords:** Aortic valve stenosis, Aortic valve replacement, TAVR, SAVR

## Abstract

**Background:**

There is limited information on the longer-term outcome after transcatheter aortic valve replacement (TAVR) with new-generation prostheses compared to surgical aortic valve replacement (SAVR). The aim of this study was to compare the mid-term outcomes after TAVR with Sapien 3 and SAVR with Perimount Magna Ease bioprostheses for severe aortic stenosis.

**Methods:**

In a retrospective study, we included patients who underwent transfemoral TAVR with Sapien 3 or SAVR with Perimount Magna Ease bioprosthesis between January 2008 and October 2017 from the nationwide FinnValve registry. Propensity score matching was performed to adjust for differences in the baseline characteristics. The Kaplan-Meir method was used to estimate late mortality.

**Results:**

A total of 2000 patients were included (689 in the TAVR cohort and 1311 in the SAVR cohort). Propensity score matching resulted in 308 pairs (STS score, TAVR 3.5 ± 2.2% vs. SAVR 3.5 ± 2.8%, *p* = 0.918). In-hospital mortality was 3.6% after SAVR and 1.3% after TAVR (*p* = 0.092). Stroke, acute kidney injury, bleeding and atrial fibrillation were significantly more frequent after SAVR, but higher rate of vascular complications was observed after TAVR. The cumulative incidence of permanent pacemaker implantation at 4 years was 13.9% in the TAVR group and 6.9% in the SAVR group (*p* = 0.0004). At 4-years, all-cause mortality was 20.6% for SAVR and 25.9% for TAVR (*p* = 0.910). Four-year rates of coronary revascularization, prosthetic valve endocarditis and repeat aortic valve intervention were similar between matched cohorts.

**Conclusions:**

The Sapien 3 bioprosthesis achieves comparable midterm outcomes to a surgical bioprosthesis with proven durability such as the Perimount Magna Ease. However, the Sapien 3 bioprosthesis was associated with better early outcome.

**Trial registration:**

ClinicalTrials.gov Identifier: NCT03385915.

## Background

Transcatheter aortic valve replacement (TAVR) with balloon-expandable [1–3] and self-expanding [4–7] bioprosthesis has proven its efficacy and safety compared to surgical aortic valve replacement (SAVR) in the treatment of aortic stenosis (AS) regardless of the operative risk. A meta-analysis of randomized controlled trials recently showed that TAVR is associated with significant reduction of all-cause mortality, a lower risk for stroke, atrial fibrillation and bleeding, but a higher risk for permanent pacemaker implantation and major vascular complications at 2 years compared to SAVR [8]. The indications for TAVR are expanding, but it is controversial whether TAVR should be performed on a larger scale because of limited data on the long-term outcome and valve durability of TAVR prostheses compared to SAVR prostheses. Similar longer-term survival after TAVR and SAVR is observed in randomized controlled trials [1, 6, 7], but studies reporting outcomes in the real-world populations have discordant findings [9–12]. Sustained valve hemodynamics and low reintervention rate is associated with the use of first-generation balloon-expandable Sapien bioprosthesis [1, 13]. However, a higher rate of structural valve deterioration leading to hemodynamic compromise was observed with the second-generation Sapien XT valve compared to the third-generation Sapien 3 valve prosthesis and the surgical valves in the PARTNER 2 trial [14]. Importantly, TAVR with different valve types and their iterations may result in discrepant outcomes and valve performance [14–16]. Therefore it is important to compare the outcomes of each TAVR prosthesis separately against SAVR prostheses with proven long-term durability [17, 18].

The third-generation balloon-expandable Sapien 3 (Edwards Lifesciences, Irvine, CA, USA) prosthesis has bovine pericardial leaflets that are attached inside a cobalt-chromium alloy frame, and unlike its predecessors (Sapien, Sapien XT, Edwards Lifesciences, Irvine, CA, USA) has an improved external layer of polyethylene terephthalate fabric seal to minimize the risk of paravalvular regurgitation along with a redesigned frame. During manufacturing, bovine pericardial leaflets undergo the same tissue processing (ThermaFix) intended to reduce the risk of leaflet calcification as in the latest generation surgical Perimount Magna Ease (Edwards Lifesciences, Irvine, CA, USA), which is regarded as the most durable surgical bioprosthesis [17].

The aim of this study was to compare the outcome after TAVR with the Sapien 3 and SAVR with the Perimount Magna Ease bovine pericardial prostheses. To our knowledge, this is the first direct comparison of these TAVR and SAVR bioprostheses.

## Methods

### Registry design

The FinnValve registry collected data on consecutive patients who underwent TAVR or SAVR with a bioprosthesis for AS at all Finnish university hospitals (Helsinki, Kuopio, Oulu, Tampere, Turku) between January 2008 and October 2017. Inclusion criteria were age > 18 years, primary aortic valve procedure with a bioprosthesis (TAVR or SAVR) for AS with or without associated coronary revascularization. The exclusion criteria were any prior aortic valve procedure, concomitant intervention for other valve or ascending aorta, active endocarditis and a procedure for aortic valve regurgitation. The study protocol was approved by the local Institutional Review Boards in all participating centres. Data was retrospectively collected in a dedicated electronic case report system by physicians and trained research nurses. Data on mortality was obtained from the Finnish Population Register Centre and data on cardiovascular interventions was retrieved from the registry of the Finnish National Institute for Health and Welfare. The follow-up was complete, except for those few patients not residing in Finland follow-up was truncated at hospital discharge. The study followed the Strengthening the Reporting of Observational Studies in Epidemiology guidelines [19].

### Patients and outcomes

**Fig. 1:**
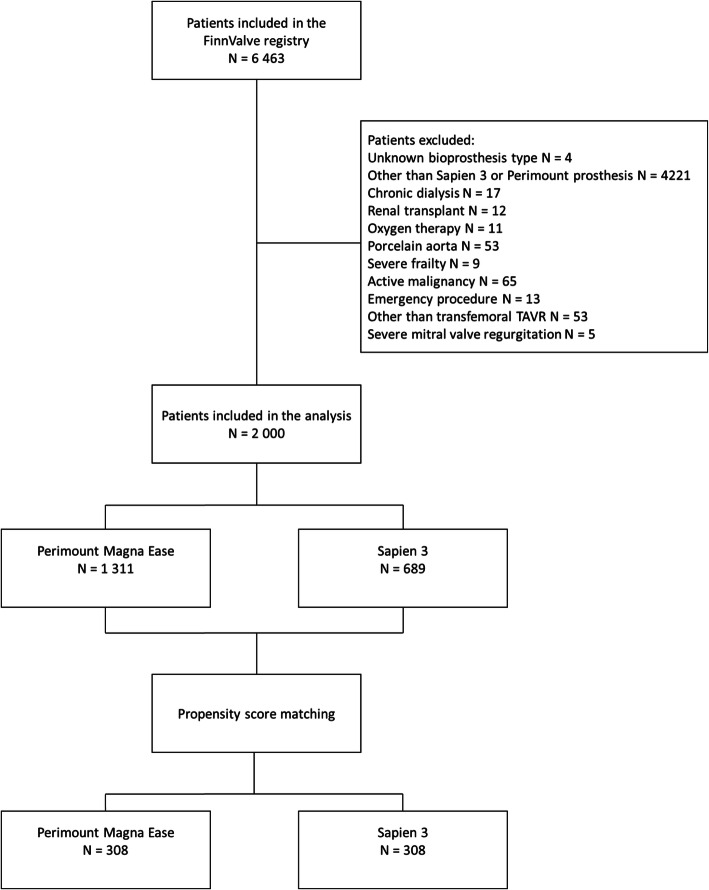
Study flow-chart

Only patients who underwent transfemoral TAVR with Sapien 3 or SAVR with Perimount Magna Ease were included in this analysis. The choice between TAVR and SAVR was based on individual assessment by the local Heart Team. Patients in the TAVR group and concomitant coronary artery disease (CAD) underwent revascularization based on the discretion of the treating physician. Patients who underwent an emergency procedure or with associated severe clinical conditions were excluded (Fig. 1). The primary outcomes were in-hospital and 4-year all-cause mortality. The secondary outcomes were stroke, atrial fibrillation, permanent pacemaker implantation, major vascular complications, acute kidney injury, dialysis, moderate or severe paravalvular regurgitation, severe bleeding, reoperation for bleeding, red blood cell transfusion, annular or aortic rupture/dissection, conversion to cardiac surgery, coronary artery occlusion, deep sternal wound infection, postoperative intra-aortic balloon pump or extracorporeal membrane oxygenation, ventricular wall injury and length of index hospitalization. Late secondary outcomes were permanent pacemaker implantation, coronary revascularization, prosthetic valve endocarditis and reoperation on the implanted aortic bioprosthesis.

The European System for Cardiac Operative Risk Evaluation (EuroSCORE) II criteria were applied for the definition of baseline variables and for surgical risk stratification [20]. Surgical risk was estimated also according to the Society of Thoracic Surgeons Predicted Risk of Mortality (STS-PROM) score. Coronary artery disease was defined as a ≥ 50% stenosis in a main coronary artery. Severe frailty was defined as Geriatric Status Scale 2–3 [21]. Stroke and major vascular complications were defined according to Valvular Academic Consortium 2 (VARC-2) [22] criteria and severe bleeding according to European Coronary Artery Bypass Grafting (E-CABG) bleeding scores 2–3 [23], i.e. transfusion of more than 4 units of red blood cells and/or reoperation for bleeding. Acute kidney injury was defined according to the KDIGO definition criteria [24].

### Statistical analysis

Statistical analyses were performed using SAS v. 9.2 (SAS Institute Inc., Cary, NC) and SPSS v. 25.0 (IBM Corporation, New York, USA) statistical softwares. Data is presented as means ± standard deviation for continuous variables, and as counts and percentages for categorical variables. Mann-Whitney’s test was used to compare continuous variables, and the chi -square and Fisher’s exact tests were used to compare the categorical variables in the unmatched cohorts. A propensity score was calculated with a non-parsimonious logistic regression model including the following variables**:** age, gender, body mass index, diabetes, atrial fibrillation, extracardiac arteriopathy, chronic lung disease, hemoglobin, estimated glomerular filtration rate, stroke, pre-existing pacemaker, previous cardiac surgery, previous percutaneous coronary intervention, coronary artery disease, number of diseased coronaries, recent myocardial infarction, New York Heart Association class IV symptoms, acute heart failure or critical preoperative state, urgent procedure, left ventricular ejection fraction, systolic pulmonary pressure, mitral valve regurgitation, frailty and inactive malignancy. One-to-one propensity score matching was performed using the nearest-neighbour method and a caliper width of 0.2 of the standard deviation of the logit of the propensity score. Baseline variables and in-hospital outcomes in the matched population were compared with paired t-test and the McNemar test. Kaplan-Meier method with Klein-Moeschberger log-rank test was used to estimate late mortality. The risk for late adverse events was calculated with competing risk analysis and comparisons were performed using the Gray’s k-sample test for equality of cumulative incidence functions. Hazard ratios were calculated with their 95% confidence intervals (CI). *P* < 0.05 was considered statistically significant.

## Results

### Study population

A total of 6463 patients were included in the FinnValve registry, and after exclusion of 4463 patients (Fig. 1), 2000 patients were subjects for the present analysis. Among them, 689 underwent TAVR with Sapien 3 bioprosthesis and 1311 patients underwent SAVR with Perimount Magna Ease prosthesis. The mean follow-up of the overall series and of the TAVR and SAVR cohorts was 3.6 ± 2.1, 2.4 ± 1.0 and 4.2 ± 2.1 years, respectively. The patients in the TAVR cohort were older (81.3 ± 6.4 vs. 74.0 ± 6.9 years), had more often co-morbidities and higher surgical risk based on the EuroSCORE II and STS-PROM scores (Table 1). After propensity score matching, 308 pairs with balanced baseline variables were identified (Table 1). The standardized difference after matching was < 0.1 for all baseline and operative covariates except for concomitant coronary revascularization, which was more common in SAVR (27.3% vs. 4.5%) despite a similar prevalence of coronary artery disease in the cohorts. The mean STS-PROM score was 3.5 ± 2.2% in the TAVR and 3.5 ± 2.8 in the SAVR cohort (*p* = 0.918) (Table 1). The sizes of implanted prostheses are summarized in Table 2.

**Table 1:** Baseline characteristics of the unmatched and propensity score matched cohorts

	Unmatched cohort				Propensity score matched cohort			
Clinical variables	Sapien 3 (***N*** = 689)	Perimount Magna Ease (***N*** = 1311)	Standardized difference	***p***-value	Sapien 3 (***N*** = 308)	Perimount Magna Ease (***N*** = 308)	Standardized difference	***p***-value
Age, yrs	81.3 ± 6.4	74.0 ± 6.9	1.1	< 0.0001	78.8 ± 6.9	79.0 ± 5.3	−0.03	0.697
Female	365 (53.0)	556 (42.4)	0.21	< 0.0001	160 (51.9)	165 (53.6)	−0.03	0.674
BMI, kg/m^2^	27.4 ± 4.9	28.0 ± 4.8	−0.12	0.012	28.1 ± 5.2	28.0 ± 5.0	0.02	0.848
Diabetes mellitus	207 (30.0)	353 (26.9)	0.07	0.140	93 (30.2)	87 (28.2)	0.04	0.578
Atrial fibrillation	293 (42.5)	255 (19.5)	0.52	< 0.0001	102 (33.1)	99 (32.1)	0.02	0.782
Extracardiac arteriopathy	117 (17.0)	137 (10.5)	0.19	< 0.0001	49 (15.9)	41 (13.3)	0.07	0.383
Chronic lung disease	149 (21.6)	172 (13.1)	0.23	< 0.0001	65 (21.1)	62 (20.1)	0.02	0.761
Hemoglobin, g/l	125.7 ± 15.2	133.6 ± 15.1	−0.53	< 0.0001	128.7 ± 15.2	127.8 ± 15.3	0.06	0.421
eGFR, ml/m^2^/min	62.0 ± 18.5	72.6 ± 16.7	−0.60	< 0.0001	65.6 ± 18.1	66.4 ± 16.1	−0.05	0.550
History of stroke	70 (10.2)	70 (5.3)	0.18	0.0001	27 (8.8)	29 (9.4)	−0.02	0.782
Prior pacemaker	65 (9.4)	50 (3.8)	0.23	< 0.0001	20 (6.5)	19 (6.2)	0.01	0.862
Previous cardiac surgery	110 (16.0)	24 (1.8)	0.51	< 0.0001	17 (5.5)	18 (5.8)	−0.01	0.847
Prior PCI	140 (20.3)	130 (9.9)	0.29	< 0.0001	47 (15.3)	40 (13.0)	0.07	0.370
Coronary artery disease	181 (26.3)	563 (42.9)	−0.36	< 0.0001	102 (33.1)	97 (31.5)	0.04	0.665
No. of diseased vessels	0.36 ± 0.7	0.78 ± 1.1	−0.48	< 0.0001	0.47 ± 0.8	0.46 ± 0.8	0.02	0.836
Recent MI	17 (2.5)	72 (5.5)	−0.16	0.0018	9 (2.9)	9 (2.9)	0.00	1.000
NYHA class IV	82 (11.9)	94 (7.2)	0.16	0.0004	31 (10.1)	34 (11.0)	−0.03	0.696
AHF	75 (10.9)	101 (7.7)	0.11	0.017	33 (10.7)	33 (10.7)	0.00	1.000
Urgent procedure	55 (8.0)	148 (11.3)	−0.11	0.020	28 (9.1)	33 (10.7)	−0.05	0.508
Ejection fraction			0.26	< 0.0001			0.08	0.699
> 50%	499 (72.4)	1069 (81.5)			230 (74.7)	239 (77.6)		
31–50%	158 (22.9)	220 (16.8)			68 (22.1)	60 (19.5)		
21–30%	31 (4.5)	22 (1.7)			10 (3.2)	9 (2.9)		
Sys. pulmonary pressure			0.74	< 0.0001			0.09	< 0.0001
31–55 mmHg	245 (35.6)	524 (40.0)			131 (42.5)	121 (39.3)		
> 55 mmHg	75 (10.9)	92 (7.0)			34 (11.0)	39 (12.7)		
Mitral valve regurgitation			0.56	< 0.0001			0.06	0.652
Mild	255 (37.0)	278 (21.2)			116 (37.7)	107 (34.7)		
Moderate	80 (11.6)	39 (3.0)			23 (7.5)	21 (6.8)		
Concomitant coronary revascularization	29 (4.2)	511 (39.0)	−0.78	< 0.0001	14 (4.5)	84 (27.3)	−0.62	< 0.0001
EuroSCORE II, %	6.5 ± 7.1	3.4 ± 4.2	0.52	< 0.0001	5.0 ± 5.2	4.9 ± 5.9	0.02	0.752
STS-PROM, %	4.3 ± 2.9	2.6 ± 2.1	0.67	< 0.0001	3.5 ± 2.2	3.5 ± 2.8	0.01	0.918

Categorical values are reported as counts and percentages. Continuous variables are reported as mean and standard deviation. *AHF* acute heart failure (within 60 days before procedure or critical preoperative state), *BMI* body mass index, *eGFR* estimated glomerular filtration rate, *EuroSCORE* European System for Cardiac Operative Risk Evaluation, *MI* myocardial infarction within 90 days before procedure, *NYHA* New York Heart Association, *PCI* percutaneous coronary intervention, *STS-PROM* Society of Thoracic Surgeons Predicted Risk of Mortality

**Table 2:** Prosthesis sizes in the unmatched cohorts

Size	Sapien 3 (***N*** = 689)	Size	Perimount Magna Ease (***N*** = 1311)
20 mm	2 (0.3)	19 mm	35 (2.7)
23 mm	221 (32.1)	21 mm	320 (24.4)
26 mm	256 (37.2)	23 mm	551 (42.0)
29 mm	206 (29.9)	25 mm	296 (22.6)
		27 mm	95 (7.2)
		29 mm	10 (0.8)

Categorical values are reported as counts and percentages

### Early outcomes

The early outcomes of the unmatched TAVR and SAVR cohorts are summarized in Table 3.

**Table 3:** Outcomes in the unmatched and propensity score matched cohorts

	Unmatched cohort			Propensity score matched cohort		
Outcomes	Sapien 3 (***N*** = 689)	Perimount Magna Ease (***N*** = 1311)	***p***-value	Sapien 3 (***N*** = 308)	Perimount Magna Ease (***N*** = 308)	***p***-value
In-hospital death	8 (1.2)	26 (2.0)	0.177	4 (1.3)	11 (3.6)	0.092
Stroke	9 (1.3)	48 (3.7)	0.003	1 (0.3)	11 (3.6)	0.006
Vascular complications			< 0.0001			< 0.0001
Minor	18 (2.6)	0		8 (2.6)	0	
Major	58 (8.4)	13 (1.0)		29 (9.4)	2 (0.6)	
Annulus rupture	2 (0.3)	0		1 (0.3)	0	
Aortic dissection/rupture	2 (0.3)	7 (0.5)	0.727	1 (0.3)	1 (0.3)	1.000
Coronary ostium occlusion	2 (0.3)	2 (0.2)	0.612	1 (0.3)	2 (0.6)	1.000
Acute kidney injury stages 2–3	5 (0.7)	72 (5.5)	< 0.0001	1 (0.3)	24 (7.8)	< 0.0001
Postoperative dialysis	2 (0.3)	20 (1.5)	0.012	0	7 (2.3)	0.015
Moderate/severe paravalvular regurgitation	8 (1.2)	10 (0.8)	0.370	6 (1.9)	4 (1.3)	0.754
Severe bleeding*	14 (2.1)	282 (21.9)	< 0.0001	4 (1.3)	88 (29.0)	< 0.0001
Reoperation for bleeding	14 (2.0)	129 (9.8)	< 0.0001	7 (2.3)	33 (10.7)	< 0.0001
Red blood cell transfusion, units	0.3 (1.1)	2.6 (3.4)	< 0.0001	0.27 (1.0)	3.2 (3.5)	< 0.0001
Postoperative IABP or ECMO	0	11 (0.8)	0.020	0	3 (1.0)	0.249
Atrial fibrillation	269 (39.0)	733 (55.9)	< 0.0001	102 (33.1)	200 (64.9)	< 0.0001
Permanent pacemaker implantation	52 (7.5)	47 (3.6)	< 0.0001	28 (9.1)	16 (5.2)	0.064
Hospital stay, days	4.0 ± 3.4	7.7 ± 5.5	< 0.0001	4.1 ± 3.7	8.4 ± 6.8	< 0.0001

Categorical values are reported as counts and percentages. Continuous variables are reported as mean and standard deviation. *ECMO* extracorporeal membrane oxygenation, *IABP* intra-aortic balloon pump; ^*^ = transfusion of more than 4 units of red blood cells and/or reoperation for bleeding

In the propensity matched cohorts, TAVR had a numerically lower in-hospital mortality (1.2% vs. 3.6%, *p* = 0.092) compared to SAVR (Table 3). Moreover, postoperative stroke was significantly less frequent after TAVR (0.3% vs. 3.6%, *p* = 0.006). A trend towards a higher need of permanent pacemaker implantation early after the procedure was observed in the Sapien 3 group. The incidence of moderate or severe paravalvular regurgitation was similar in both cohorts. TAVR was associated with lower rates of postoperative atrial fibrillation, acute kidney injury and severe bleeding compared to SAVR (Table 3). Major vascular complications were significantly more frequent in the TAVR cohort. Annular rupture occurred in one patient after Sapien 3 implantation (Table 3).

### Mid-term outcomes after procedures

**Fig. 2:**
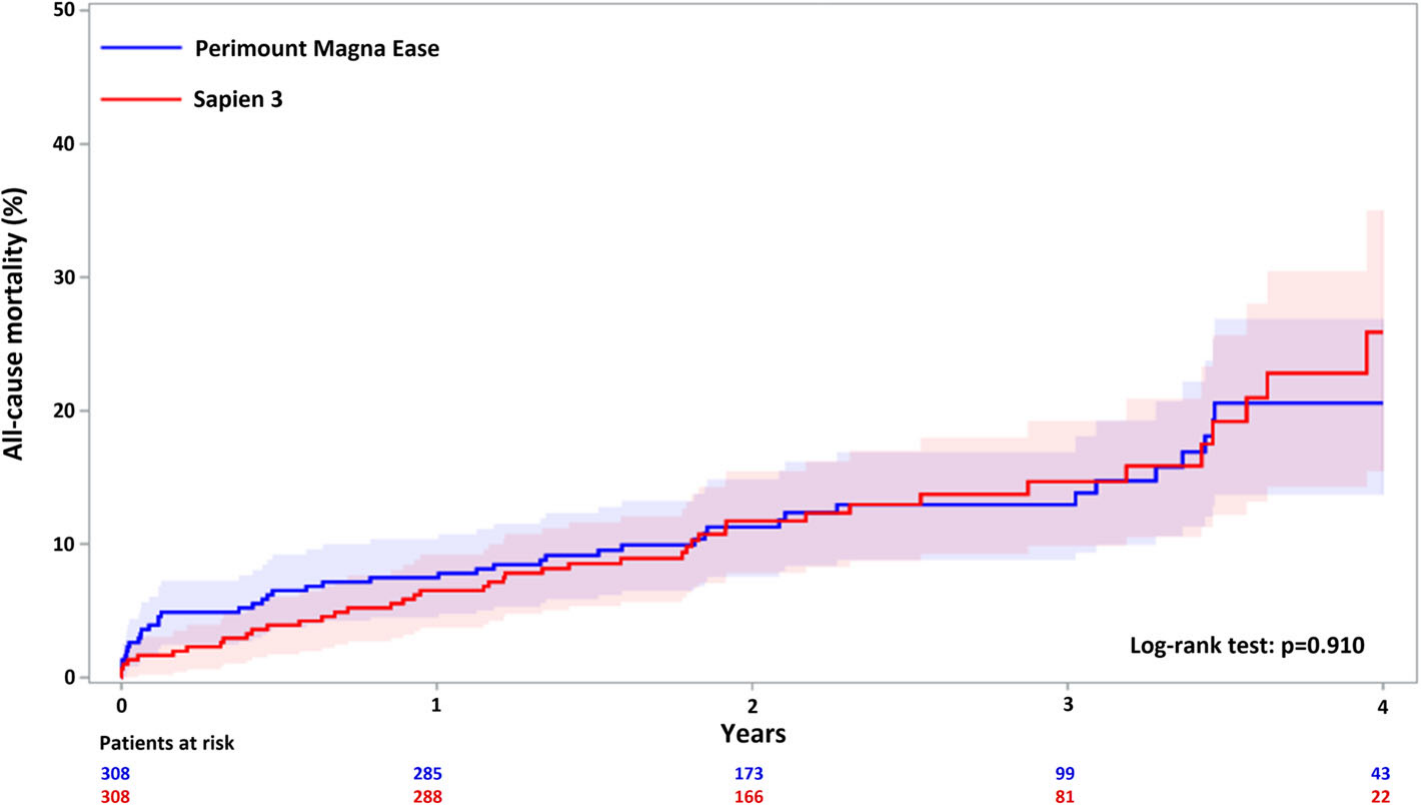
Kaplan-Meier estimate of all-cause mortality after aortic valve replacement with Sapien 3 and Perimount Magna Ease bioprostheses in the propensity score matched cohorts. *P*-value is from the Klein-Moeschberger log-rank test

In the matched cohorts, Kaplan-Meier estimate of all-cause mortality was 7.5 and 6.5% at 1-year, 11.3 and 11.7% at 2-years, 12.9 and 14.7% at 3-years, 20.6 and 25.9% at 4-years in the SAVR and TAVR cohorts, respectively (HR 0.96; 95% CI 0.63–1.46; *p* = 0.910) (Fig. 2). At 4-years, the cumulative incidence of permanent pacemaker implantation was higher after TAVR (13.9% vs. 6.9%; HR 2.16; 95% CI 1.27–3.68). TAVR was associated with similar rates of late coronary revascularization (1.5% vs. 1.4%; HR 0.76; 95% CI 0.17–3.43), prosthetic valve endocarditis (0.6% vs. 0.5%; HR 1.02; 95% CI 0.06–16.10) and repeat aortic valve intervention (0.4% vs. 0.4%; HR 1.02; 95% CI 0.06–16.14) compared to SAVR.

In the matched groups, one patient in the TAVR group underwent aortic valve reintervention for structural valve deterioration and one patient in the SAVR group for paravalvular regurgitation. Additionally, the indications for reoperation in the unmatched cohorts were structural valve deterioration (1 patient with Sapien 3, 1 patient with Perimount), paravalvular regurgitation (5 patients with Perimount), and endocarditis (1 patient with Perimount).

## Discussion

The main findings of our study are the following: 1) patients treated for severe AS with the transfemoral TAVR with the Sapien 3 bioprosthesis had similar mid-term mortality compared to patients who underwent SAVR with the Perimount Magna Ease bioprosthesis; 2) the risk for coronary revascularization, repeat aortic valve intervention and prosthetic valve endocarditis at 4 years was low and similar with both bioprostheses; 3) the Sapien 3 was associated with a higher cumulative rate of permanent pacemaker implantation than the Perimount Magna Ease bioprosthesis; 4) procedural safety in terms of stroke, atrial fibrillation, kidney injury and bleeding favoured TAVR, whilst lower rate of major vascular complications was observed with SAVR.

We hypothesised that unbiased evaluation on the outcomes after TAVR and SAVR could be feasible by including only the Sapien 3 and the Perimount Magna Ease bioprostheses, because the bioprostheses share some technological features such as bovine pericardial leaflets utilizing similar anti-calcification processes during manufacturing. Furthermore, the Perimount Magna Ease demonstrated an excellent durability among current surgical bioprostheses [17].

Our study showed that TAVR with the Sapien 3 prosthesis resulted in similar survival compared to SAVR with the Perimount Magna Ease at 4-years. Comparable mid-term outcomes between TAVR and SAVR were achieved in randomized controlled trials [1, 6, 7], but strict selection of the patients does not allow generalization of the results into real-life AS patients undergoing invasive treatment. Indeed, several observational studies have shown inferior mid-term outcomes after TAVR compared to SAVR. The findings from the OBSERVANT registry of 1300 matched patients undergoing TAVR with first/second generation prostheses and SAVR showed that TAVR was associated with higher all-cause mortality (44.5% vs. 35.8%) at 5-years. Mortality rate in the OBSERVANT registry exceeded our 4-year mortality already after 2.5 years [11]. Markedly inferior 5-year survival after TAVR was observed also in the analysis from the French Medical Information System database, with all-cause death of 52% after TAVR and 37% after SAVR in the matched patient populations [9]. There are few possible explanations for such different mid-term outcomes between the studies. Firstly, the Sapien 3 bioprosthesis carried a decreased risk of structural valve deterioration compared to its predecessor the Sapien XT valve, and performed similarly as the surgical valves in the PARTNER 2 study [14]. In addition, a propensity score matched study combining data from the SOURCE XT and the SOURCE 3 registries showed improved survival with Sapien 3 compared to Sapien XT valves [15]. A low incidence (1.9%) of moderate paravalvular regurgitation with Sapien 3 in our study was in concordance with other studies, and potentially impacted the outcomes [25]. Secondly, including different generations of balloon-expandable and self-expanding TAVR prostheses in previous observational studies may have introduced a significant bias [16].

The question of valve durability is becoming more relevant as TAVR is adopted for lower- risk patients. Several studies showed reasonable durability of surgical bioprosthesis up to 15–20 years after SAVR [17], but variable definitions used for structural valve deterioration in surgical prostheses makes benchmarking for transcatheter valves difficult [26]. The incidence of structural valve deterioration cannot be estimated in our study since a comprehensive echocardiographic follow-up data was not available and solely reintervention rate inevitably leads to a major underestimation of its true incidence. This clearly limits the interpretation of our results regarding the durability of the Sapien 3 prosthesis. However, the need for reintervention for aortic valve complications was very low in both cohorts.

The cumulative incidence of permanent pacemaker implantation in the Perimount group remained low and stable, while in the Sapien 3 group permanent pacemaker implantation was increasingly needed along the study period. Since pacing after TAVR may have long-term consequences for the patient [27], we should aim to reduce the risk for permanent pacemaker implantation by adopting higher implantation technique and avoiding excess oversizing with the Sapien 3 prosthesis [28, 29].

The prevalence of coronary artery disease was similar in the matched cohorts, but concomitant coronary revascularization was performed only in 14% of the patients with coronary artery disease in the TAVR group compared to 87% revascularization rate in patients with coronary artery disease in the SAVR group. Complete revascularization during SAVR is recommended to avoid postoperative left ventricular systolic dysfunction and excess mortality after surgery [30], but the best revascularization strategy during TAVR is not established yet, which most likely explains the lower revascularization rate in the TAVR group. Interestingly, such a low rate of coronary revascularization at the time of procedure did not expose patients undergoing TAVR to an increased need of revascularization at 4-years with similar mortality rate compared to SAVR. However, we have to interpret this finding with caution because the increased risk related to coronary artery disease in the TAVR patients is driven by its severity [31], and our definition criteria did not capture patients only with the most severe coronary artery disease.

Procedural safety is one of the major concerns in the decision-making process. The present findings indicate that TAVR with balloon-expandable Sapien 3 is safe with very low rates of annular rupture and coronary obstruction. Furthermore, TAVR was associated with lower incidence of stroke, acute kidney injury, atrial fibrillation and bleeding compared to SAVR. However, the rate of major vascular complications was still higher in TAVR compared to SAVR. This favourable safety profile of TAVR over SAVR is in alignment with current knowledge [8].

### Limitations

The retrospective nature is the major limitation of this study. Secondly, the mean follow-up in the TAVR cohort was shorter than in the SAVR cohort. Third, comparative analysis of the TAVR and SAVR cohorts was based on propensity score matching and unrecognized confounders might have had an impact on the results. Finally, the lack of complete echocardiographic follow-up prevented an analysis of structural valve deterioration which might have occurred in these cohorts.

## Conclusions

In this nationwide study, transfemoral TAVR with Sapien 3 prosthesis achieved similar mid-term outcomes with better procedural safety compared to SAVR with Perimount Magna Ease bioprosthesis.

## Data Availability

The datasets used during the current study are not available due to legal restrictions.

## Abbreviations

EuroSCORE: European System for Cardiac Operative Risk Evaluation
PARTNER: Placement of Aortic Transcatheter Valves
SAVR: Surgical aortic valve replacement
STS-PROM: Society of Thoracic Surgeons Predicted Risk of Mortality
TAVR: Transcatheter aortic valve replacement
